# ASSOCIATION OF CHILDHOOD RESIDENTIAL CHANGE WITH LATER LIFE MEMORY TRAJECTORIES IN THE HEALTH AND RETIREMENT STUDY

**DOI:** 10.1093/geroni/igae098.4337

**Published:** 2024-12-31

**Authors:** Abbey Hamlin, Dana Alhasan, Helen Meier, Mark Manning, Xuexin Yu, Sirena Gutierrez, Noah Webster

**Affiliations:** University of Texas at Austin, Austin, Texas, United States; University of North Carolina, Charlotte, Charlotte, North Carolina, United States; Institute for Social Research, Institute for Social Research, Michigan, United States; Oakland University, Rochester, Michigan, United States; University of Michigan, Ann Arbor, Michigan, United States; University of California, San Francisco, San Francisco, California, United States; University of Michigan, Ann Arbor, Michigan, United States

## Abstract

Childhood residential change may affect later life memory, although few studies have examined this relationship, and research in diverse racial/ethnic samples is sparse. Data were from the US Health and Retirement Study. Childhood residential change was measured by the number of residences before the age of 16 (0-1, 2, 3, 4 or more) (N=4020). Moving due to financial difficulties before 16 years old was categorized as yes versus no (N=4005). Memory function was measured using composite memory z-scores incorporating direct and proxy assessments from 1996-2016. We utilized mixed-effects linear regression models with subject-specific random slopes and intercepts adjusting for sociodemographic characteristics to estimate associations between residential change and memory, by race/ethnicity and parental education. The mean age was 57.5±5.9 years, 82.1% self-identified as non-Hispanic White, 12.7% as non-Hispanic Black, and 5.2% as Other/Unknown. Fewer moves during childhood were associated with a higher initial average memory level, but a faster rate of memory decline. Moving due to financial difficulties during childhood was not associated with initial memory levels, but was associated with faster memory decline. We did not observe effect modification by race/ethnicity or parental education. Results suggest that childhood residential change and the reasons for residential change may contribute to later life memory function and decline.

